# Promoting plasmonic photocatalysis with ligand-induced charge separation under interband excitation†

**DOI:** 10.1039/d3sc02167j

**Published:** 2023-07-27

**Authors:** Ben Roche, Tamie Vo, Wei-Shun Chang

**Affiliations:** a Department of Chemistry and Biochemistry, University of Massachusetts Dartmouth North Dartmouth Massachusetts 02747 USA wchang2@umassd.edu

## Abstract

Plasmonic nanoparticles have been demonstrated to enhance photocatalysis due to their strong photon absorption and efficient hot-carrier generation. However, plasmonic photocatalysts suffer from a short lifetime of plasmon-generated hot carriers that decay through internal relaxation pathways before being harnessed for chemical reactions. Here, we demonstrate the enhanced photocatalytic reduction of gold ions on gold nanorods functionalized with polyvinylpyrrolidone. The catalytic activities of the reaction are quantified by *in situ* monitoring of the spectral evolution of single nanorods using a dark-field scattering microscope. We observe a 13-fold increase in the reduction rate with the excitation of d-sp interband transition compared to dark conditions, and a negligible increase in the reduction rate when excited with intraband transition. The hole scavenger only plays a minor role in the photocatalytic reduction reaction. We attribute the enhanced photocatalysis to an efficient charge separation at the gold–polyvinylpyrrolidone interface, where photogenerated d-band holes at gold transfer to the HOMO of polyvinylpyrrolidone, leading to the prolonged lifetime of the electrons that subsequently reduce gold ions to gold atoms. These results provide new insight into the design of plasmonic photocatalysts with capping ligands.

## Abstract 2

Polyvinylpyrrolidone, a capping ligand adsorbed on gold nanorods, induces electron–hole separation to prolong the hot carrier lifetime and increase the efficiency of plasmonic photocatalysis under interband excitation.

## Graphical abstract

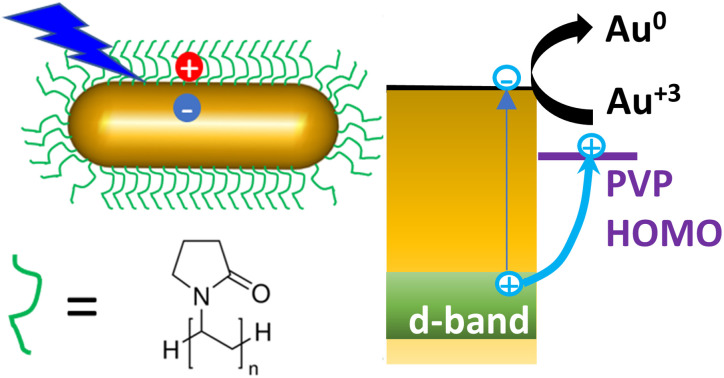

## Introduction

An ideal photocatalyst requires efficient photon absorption to create highly energetic carriers to enable reactions with a high activation energy.^1,2^ Plasmonic nanoparticles absorb photons more efficiently at plasmon frequencies than other materials.^3^ Additionally, the tunability of the plasmon energy by the morphology and composition of nanostructures allows harvesting the photons from UV to IR regions.^4–7^ Upon photon excitation, plasmons decay non-radiatively into highly energetic electron–hole pairs that can subsequently drive chemical reactions.^8–16^ Due to these excellent properties, plasmonic nanoparticles have been demonstrated as photocatalysts or photoelectrocatalysts to drive various reactions, such as NH_3_ decomposition,^17,18^ CO_2_ reduction,^19–26^ hydrogen generation,^27–29^ and NH_3_ formation.^30–33^

Photoexcitation of plasmonic nanoparticles through interband and intraband transitions generates hot carriers with various energies.^34–37^ For gold (Au) nanoparticles, photons with energy higher than the interband threshold (2.4 eV) excite the electrons from the d-band to the sp-band of the metal, creating hot holes and warm electrons populating at the d-band and near the Fermi level, respectively. On the other hand, excitation of surface plasmon resonances promotes electrons near the Fermi level to the unoccupied states within the conduction band, producing hot electrons above the Fermi level and warm holes near the Fermi level^34–37^ (Fig. S1†). These highly energetic carriers produced by the photoexcitation of plasmonic nanoparticles have been shown to catalyze chemical reactions with high activation barriers.^17–33^ However, the lifetime of these plasmon-induced hot carriers, limited by electron–hole recombination and electron–electron scattering, is less than 100 fs,^34,38–44^ which is several orders of magnitude shorter than the chemical reaction time. As a result, extracting the hot carriers before their recombination is critical to the realization of plasmonic photocatalysis.

Several strategies were developed to prolong the hot carrier lifetime by inducing charge separation states at the heterojunction to counteract the ultrafast recombination of electrons and holes in the metal.^45^ Hybrid nanostructures were created by depositing various materials on Au nanostructures, including semiconductors and molecular adsorbates.^46–53^ Exciting the surface plasmon resonances of Au/semiconductor nanostructures, the electrons of the metal transfer to the conduction band of the semiconductors while the holes stay in the metal.^46–48^ In addition, hot holes transfer to the p-GaN with electrons in the Au nanostructures under interband excitation.^49^ In hybrid nanostructures composed of plasmonic nanoparticles and surface adsorbates, the plasmon excitation directly excites the electrons from the Fermi level of the metal to the LUMO of the molecular adsorbates, leading to charge separation.^50–53^ An efficient charge separation was also observed in Au nanoparticles with hole scavengers under interband excitation.^54–56^ Hence, maximizing the charge separation of hybrid nanostructures with the type of excitation will advance our knowledge for the design of plasmonic photocatalysts.

Colloidal nanoparticles are stabilized using capping ligands to avoid aggregation of nanoparticles in the solution. Capping ligands are widely accepted as surface-blocking ligands that inhibit chemical reactions. Recently, Wei has demonstrated the growth of small Au seeds into hexagonal prisms with polyvinylpyrrolidone (PVP) ligands in the presence of hole scavengers under the excitation of the surface plasmon resonances of Au nanoseeds.^57,58^ The anisotropic growth arises from photochemical reduction of Au^3+^ ions at the perimeter of the nanoprism, where PVP ligands are specifically adsorbed. After creating the electron–hole pairs by plasmon excitation, the holes were removed by the hole scavenger, methanol, while the hot electrons were stabilized by the positively charged PVP. Therefore, the PVP ligand plays an important role in the plasmon-induced Au^3+^ reduction reactions rather than serving as a surface-blocking ligand. This strategy of anisotropic growth of nanoseeds into nanoprisms has been utilized to fabricate periodic nanoprisms with various morphologies.^59,60^ However, the interband and intraband transitions for the Au nanoseeds are spectrally overlapped. It is difficult to distinguish whether interband or intraband transitions promote Au^3+^ reduction in the presence of PVP ligands. Furthermore, PVP could play a different role in photoinduced growth in addition to stabilizing the hot electrons and AuCl_4_^−^. Understanding the underlying mechanism of reduction reactions with PVP is critical to promoting plasmonic catalysis using capping ligands that could potentially induce charge separation.

Here, we utilized PVP-functionalized Au nanorods (AuNRs) as seeds to investigate photoinduced Au^3+^ reduction by single-particle dark-field scattering (DFS) spectroscopy. AuNRs allow for separating the interband and intraband transitions as the longitudinal plasmon modes are located at an energy lower than 2.0 eV, which is below the interband onset of Au. Single-particle measurements resolve sample heterogeneity to unambiguously quantify the growth rate of individual AuNRs.^61^ We found that the growth rate of AuNRs in the presence of PVP increases more than ten times under interband transition compared to that in the dark, while intraband excitation does not enhance the growth rate. Hole scavengers only have a minor contribution to the enhancement of the Au^3+^ reduction reaction mediated by PVP. Additionally, the reduction reaction preferentially occurs at the location of PVP. These results reveal that d-band holes transfer to PVP under interband transition, while the high density of warm electrons in Au reduces Au^3+^ to promote AuNR growth. Electrons and holes generated by intraband excitation suffer from ultrafast recombination due to the lack of charge separation, prohibiting the reduction of Au^3+^. Our findings provide insight into the usage of capping ligands to induce charge separation to promote plasmonic catalysis.

## Results and discussion

The plasmon-catalyzed Au^3+^ reduction reaction was performed using PVP-functionalized AuNRs (21.6 ± 3.1 × 53.5 ± 5.9 nm, Fig. S2†) as seeds, and the reaction kinetics was probed by single-particle DFS spectroscopy. AuNRs with an aspect ratio of 2.5 ± 0.4 exhibit the longitudinal and transverse modes at 700 and 520 nm, respectively, in an aqueous solution (Fig. S3†). As a result, it is possible to separately excite the intraband and interband transitions by photons with wavelengths longer than 600 nm and shorter than 500 nm, respectively. Additionally, the peak energy of AuNRs blueshifts with a smaller aspect ratio and the intensity scales with their size. One can measure the spectral evolution of AuNRs by single-particle DFS spectroscopy to probe their morphology change without using high-resolution electron microscopy. Furthermore, single-particle measurements enable acquiring the spectra of hundreds of AuNRs during the reaction (see the Method section in the ESI† and the discussion below) to obtain the statistical information, which is inaccessible by electron microscopy. Finally, quantifying the photocatalytic efficiency of Au^3+^ reduction as a function of the aspect ratio of AuNRs can be achieved by single-particle spectroscopy with “one” measurement, as AuNRs exhibit a heterogeneous distribution of the aspect ratio (Fig. S2†).

**Fig. 1:**
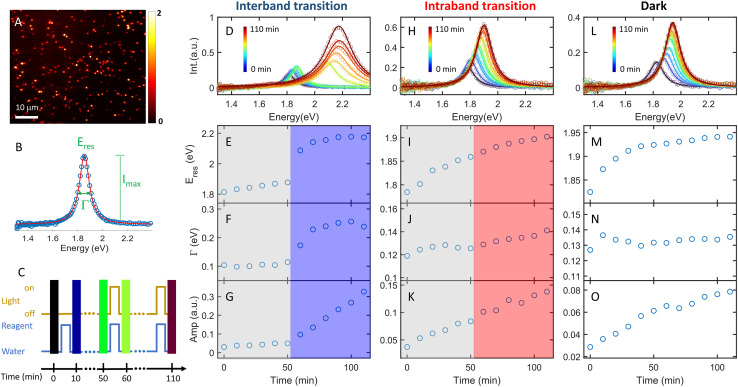
*In situ* monitoring of single AuNR growth under photoexcitation and in the dark. (A) Hyperspectral images of AuNRs in water. The scale bar is 10 μm. (B) Scattering spectrum (blue symbols) of a single AuNR fit to a Lorentzian function (red) to obtain plasmon energy (*E*_res_), full width at half maximum (*Γ*), and peak intensity (*I*_max_). (C) Sequence of the experiment. The blue line represents the injection of water (valley) and the reagent (peak) into the cell. The golden line indicates the light on (peak) and off (valley). The color bars are the sequence of hyperspectral images. The time duration of the peak for the blue and golden lines is 10 minutes. (D) Spectral evolution of a representative AuNR during the Au^3+^ reduction reaction with interband excitation. (E–G) Temporal evolution of *E*_res_ (E), *Γ* (F), and the amplitude (G) of the scattering spectra shown in (D). The grey and blue shadows indicate the blue light off and on, respectively. (H) Spectral evolution of a representative AuNR during the Au^3+^ reduction reaction with intraband excitation. (I–K) Temporal evolution of *E*_res_ (I), *Γ* (J), and the amplitude (K) of the scattering spectra shown in (H). The grey and red shadows indicate the red light off and on, respectively. (L) Spectral evolution of a representative AuNR during the Au^3+^ reduction reaction in the dark. (M–O) Temporal evolution of *E*_res_ (M), *Γ* (N), and the amplitude (O) of the scattering spectra shown in (L).

To perform the photocatalytic reduction reaction, PVP functionalized AuNRs were spin-cast on a silanized coverslip that was assembled into a flow cell with another glass slide spaced with a parafilm (Method section in the ESI†). The flow cell was mounted on a DFS microscope with hyperspectral imaging capacity. Hyperspectral imaging allows one to acquire the scattering spectra of hundreds of single AuNRs in a few minutes (Fig. 1A). The recorded spectrum (blue symbol in Fig. 1B) was fit with a Lorentzian function (red line in Fig. 1B) to obtain the peak energy (*E*_res_), full width at half maximum (*Γ*), maximum intensity (*I*_max_), and amplitude 
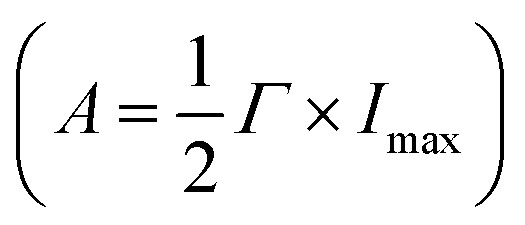
. A series of hyperspectral images of the same AuNRs were acquired at various reaction times (*t*) with and without photoexcitation using the experimental sequence shown in Fig. 1C and S4† (see the section of the Method in the ESI† for the detailed procedure). A reagent containing 3 μM HAuCl_4_, 15 μM PVP, and 2.9 M ethanol was introduced into the cell and incubated for 10 minutes to allow the reduction of Au^3+^ (peak of the blue line). Subsequently, the reaction was stopped by flowing DI water to the cell to completely remove the reagent (valley of the blue line), and the hyperspectral images of AuNRs were acquired (color bars). We defined the reaction time as the total length of the incubation time in the reagent. The spectral evolution of AuNRs was obtained at *t* = 0–50 min without light illumination and *t* = 60–110 min under photoexcitation (golden line). The excitations of interband and intraband transitions were realized by placing a blue filter (500 nm short-pass filter) and a red filter (610 nm long-pass filter) after the lamp, respectively. Two IR filters were also inserted in the excitation path to avoid heating the aqueous solution by absorbing IR photons.

The AuNRs exhibit fast growth under interband excitation, while the intraband transition has a minimal contribution. Fig. 1D shows the spectral evolution of a representative AuNR with the interband excitation. The time-dependent *E*_res_, *Γ*, and amplitude are shown in Fig. 1E, F, and G, respectively. At *t* = 0–50 min, the spectra show a small blueshift in *E*_res_ (Fig. 1E) and a slight increase in the amplitude (Fig. 1G), suggesting a minor growth of the AuNR into a smaller aspect ratio in the dark. In stark contrast, the spectra blueshift to ∼2.2 eV with a momentous increase in the amplitude at *t* = 60–110 min, indicating an efficient growth of AuNRs under the interband excitation. The AuNR was reshaped into a large isotropic nanoparticle (Fig. S5†). The spectral evolution of a AuNR excited by intraband transition shows a gradual shift to higher energy and a small increase in the amplitude (Fig. 1H–K). Compared to a control experiment where the reaction was carried out in the dark at *t* = 0–110 min, the spectral evolution of the AuNR in the dark (Fig. 1L–O) is similar to that with intraband excitation. This result indicates a negligible contribution of the intraband transition to AuNR growth in the presence of the PVP ligands.

**Fig. 2:**
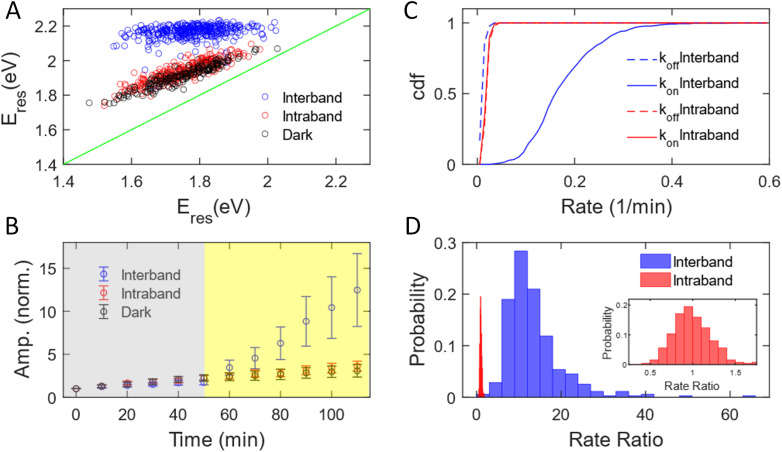
Growth of AuNRs with PVP ligands under interband and intraband transition. (A) Correlation between *E*_res_ of individual AuNRs at *t* = 0 (*x*-axis) and 110 min (*y*-axis) under interband (blue) and intraband (red) transitions and in the dark (black). The blue, red, and black symbols include data from 353, 251, and 187 AuNRs, respectively. (B) Subensemble average of the normalized amplitude of AuNRs during the growth reaction under interband (blue) and intraband (red) excitations and in the dark (black). The symbols and error bars represent the mean and standard deviation of the normalized amplitudes of AuNRs at a specific reaction time. (C) Cumulative density function of *k*_on_ (solid lines) and *k*_off_ (dashed lines) under interband (blue) and intraband (red) transitions. (D) Histogram of the rate ratio for individual AuNRs excited by interband (blue) and intraband (red) transitions. (Inset) Histogram of the rate ratio for AuNRs by intraband excitation with the rate ratio at 0–2.

We have measured 353, 251, and 187 AuNRs excited by interband and intraband transitions and in the dark, respectively. The subensemble of spectral evolution of these AuNRs exhibits a behavior similar to that of the representative AuNRs shown in Fig. 1. Fig. 2A shows the correlation of *E*_res_ at *t* = 0 min and *t* = 110 min with interband (blue) and intraband (red) transitions and without illumination (black). The *E*_res_ of AuNRs at *t* = 0 min ranging from 1.5–2 eV suggests a heterogeneous distribution of the aspect ratio of AuNRs consistent with the results obtained with a scanning electron microscope (SEM, Fig. S2†). Such sample heterogeneity is beneficial for single-particle measurements because one can access the growth mechanism of AuNRs with a variety of initial AuNR morphology in one measurement.^62^ At *t* = 110 min, the *E*_res_ of all AuNRs shifts to ∼2.2 eV under interband excitation (blue dots), indicating that AuNRs transform into isotropic nanoparticles regardless of their initial aspect ratio, supported by the SEM image of the nanoparticles (Fig. S6†). Exciting intraband transition results in a blueshift of ∼50 meV for all AuNRs (red dots), suggesting a slight reshaping of AuNRs into a smaller aspect ratio. The degrees of the peak shift of AuNRs for the intraband transition and in the dark (black dots) are almost identical, indicating that the intraband transition does not provide additional energy to transform AuNRs. To obtain the statistics of the amplitudes for different AuNRs during the reaction, the time-dependent amplitude of each AuNR was normalized by its amplitude at *t* = 0 min to eliminate the variation in the scattering intensity of AuNRs due to different illumination powers in the DFS measurements between cells. Fig. 2B displays the normalized amplitude as a function of the reaction time with interband (blue) and intraband (red) excitations and without illumination (black). The AuNRs grow slightly in the dark as the averaged amplitudes at *t* = 50 min increase by a factor of 2 for these three samples (grey area). After photon illumination, the subensemble average of the normalized amplitude exhibits a stark difference under interband and intraband transitions. The normalized amplitude increases more than 13 times at *t* = 110 min by the interband excitation. In contrast, the time-dependent normalized amplitude behaves similarly for the intraband transition and in the dark, where the averaged amplitudes increase by a factor of 3 for both cases at *t* = 110 min. Combining evidence in the change of *E*_res_, normalized amplitude, and SEM images, we can conclude that the AuNRs transform into large isotropic nanoparticles under the interband transition regardless of their initial aspect ratio. No significant reshaping was observed for AuNRs in the area without illumination (Fig. S7†). The intraband transition has a minimal impact on the Au^3+^ reduction reaction because the *E*_res_ shift and amplitude change are similar between the intraband excitation and in the dark. In both cases, the AuNRs slightly grow into a smaller aspect ratio with a slight increase in volume.

The growth rate of AuNRs excited by the interband transition is 13 times higher than that under intraband transition and in the dark. We observed that the time-dependent normalized amplitude is linear to the reaction time both in the dark and with light illumination (Fig. S8†). As a result, the time-dependent amplitude was fit to a linear equation, *y* = *kt* + *a*, at *t* = 0–50 min and *t* = 50–110 min to quantify the growth rate in the dark (*k*_off_) and under illumination (*k*_on_). We define the measured growth rate as an apparent growth rate because it is quantified using the increased spectral amplitude rather than the increased volume. The fitting was performed on the normalized amplitude as a function of the reaction time for all AuNRs with both interband transition (353 AuNRs) and intraband transition (251 AuNRs). Fig. 2C shows the cumulative density function of *k*_off_ (dashed) and *k*_on_ (solid) under interband (blue) and intraband (red) transitions. The cumulative density function for *k*_off_ (0.023 ± 0.006 min^−1^) overlaps with that for *k*_on_ (0.023 ± 0.006 min^−1^) with red-light illumination (red). The identical *k*_off_ and *k*_on_ suggest that intraband excitation does not enhance the photocatalytic activity of the Au^3+^ reduction. In contrast, *k*_on_ is 0.180 ± 0.072 min^−1^, while *k*_off_ is 0.015 ± 0.006 min^−1^ under interband excitation. The increase in the growth rate by more than 10-fold suggests a much more efficient photocatalytic reduction of Au^3+^ with the interband transition. Additionally, single-particle measurements provide enriched information on the morphology-dependent growth rate. Fig. S9† shows *k*_off_ as a function of *E*_res_ at *t* = 0 min, representing the aspect ratio of AuNRs before the reaction. The anti-correlation in Fig. S9† demonstrates a higher *k*_off_ for AuNRs with a larger aspect ratio, probably because AuNRs with a higher aspect ratio possess more surface area for chemical reactions. Furthermore, we can also correlate the *k*_on_ and *k*_off_ of the same AuNRs, as shown in Fig. S10.† Under interband transition, there is no correlation between *k*_on_ and *k*_off_, suggesting that AuNRs lose their memory with blue-light illumination. Indeed, all AuNRs transformed into isotropic shapes within 10 minutes of interband excitation, regardless of the initial aspect ratio of AuNRs, as evidenced by the fact that *E*_res_ shifted to 2.0 eV for most of the AuNRs after illumination with blue light for 10 minutes, as shown in Fig. S11.† On the other hand, *k*_off_ and *k*_on_ show a positive correlation with the intraband transition, meaning that AuNRs with a higher growth rate in the dark also grow faster with red-light illumination. These results further demonstrate the benefit of single-particle spectroscopy to take advantage of sample heterogeneity to obtain more information in “one” measurement.

We further quantified the enhanced growth rate under illumination for each AuNR by defining the rate ratio as *k*_on_/*k*_off_. Fig. 2D shows the histogram of the rate ratio for single AuNRs excited by the interband (blue) and intraband (red) transitions. The large and broad distribution of the rate ratio (13.46 ± 6.94) demonstrates an enhanced growth rate of more than one order of magnitude with a significant variation between AuNRs under interband transition. In contrast, the enhanced growth rate with the intraband transition was calculated to be 0.99 ± 0.22, indicating no increased photocatalytic activity of the Au^3+^ reduction. Based on these observations, we conclude that the excitation of the interband transition is responsible for the photoreduction of Au^3+^ in the presence of PVP.

**Fig. 3:**
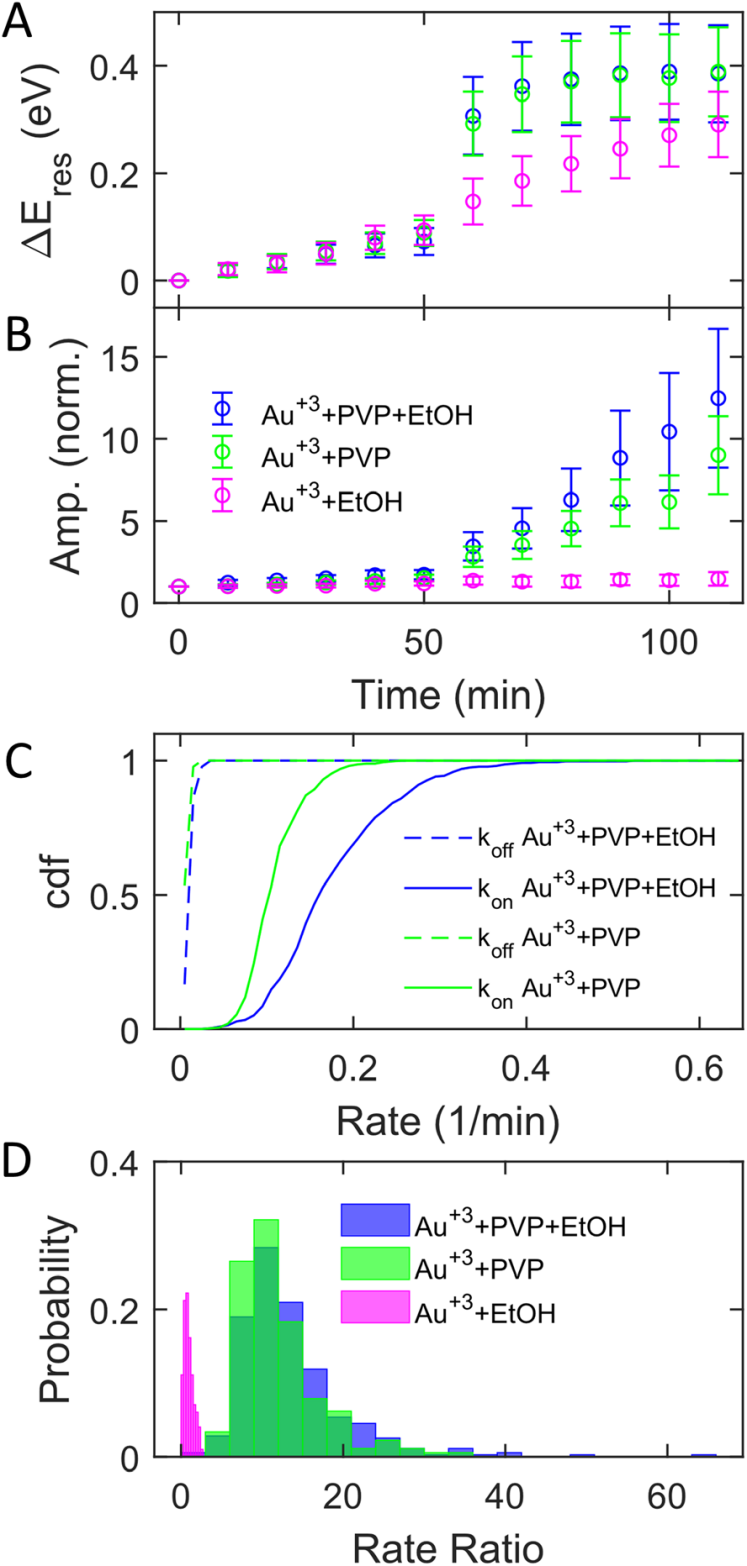
The role of PVP in the photoinduced growth of AuNRs under interband excitation. (A and B) Evolution of Δ*E*_res_ (A) and the normalized amplitude (B) in a growth solution containing Au^3+^, PVP, and ethanol (blue), Au^3+^ and PVP (green), and Au^3+^ and ethanol (magenta). The symbols and error bars represent the mean and standard deviation of the values for AuNRs, respectively. (C) Cumulative density function of *k*_on_ (solid) and *k*_off_ (dashed) with a growth solution containing Au^3+^, PVP, and ethanol (blue) and Au^3+^ and PVP (green). (D) Histogram of the rate ratio for individual AuNRs with growth solutions containing Au^3+^, PVP, and ethanol (blue), Au^3+^ and PVP (green), and Au^3+^ and ethanol (magenta).

The adsorption of PVP on AuNRs is critical to enhancing the photocatalytic reduction of Au^3+^ under interband excitation. To investigate the role of PVP, we performed experiments on AuNR growth by illuminating blue light with three different growth solutions containing (1) 3 μM HAuCl_4_, 15 μM PVP, and 2.9 M ethanol, (2) 3 μM HAuCl_4_ and 15 μM PVP, and (3) 3 μM HAuCl_4_ and 2.9 M ethanol. Fig. 3A and B show the subensemble average of the change of *E*_res_ (Δ*E*_res_(*t*) = *E*_res_(*t*) − *E*_res_(0)) and normalized amplitude as a function of reaction time, respectively. In the dark, the Δ*E*_res_ and normalized amplitude of AuNRs increase slightly with the reaction time, indicating the growth of AuNRs into smaller aspect ratios in the three reagents. The small variation (mean values within error bars) between these three cases suggests that AuNRs grow similarly in the solutions regardless of the presence of PVP and ethanol. When illuminated with blue light, AuNRs in growth solution 3 (without PVP) only show a small increase in Δ*E*_res_ and the normalized amplitude (magenta symbols). Another control experiment using AuNRs functionalized with cetyltrimethylammonium bromide (CTAB) in growth solution 3 also exhibits a slight increase in the normalized amplitude with interband excitation (Fig. S12†). These results suggest no significant growth of AuNRs in the absence of PVP under interband transition. In contrast, *E*_res_ blueshifts by 0.4 eV, and the normalized amplitude increases by a factor of 10 in growth solution 2 (without ethanol) at *t* = 110 min (green symbols). Such a large blueshift in *E*_res_ and increase in the amplitude are comparable to the results in growth solution 1 (blue symbols), indicating that all AuNRs reshape into large and isotropic nanoparticles. Based on these observations, PVP is the key to promoting the photocatalytic reduction of Au^3+^, regardless of the presence of ethanol, despite a slightly less increase in the amplitude without ethanol. Ethanol is utilized as a hole scavenger in plasmonic catalysis to remove holes and prolong the lifetime of hot electrons.^55,57^ Our results suggest that PVP ligands could serve as hole scavengers.

Further analysis of the rate ratio confirms that the enhanced growth rates with and without ethanol are comparable under interband excitation in the presence of PVP. Fig. 3C shows *k*_off_ (dashed) and *k*_on_ (solid) in growth solutions 1 (blue) and 2 (green). Averaging from 353 (blue) and 355 (green) AuNRs, *k*_off_ = 0.015 ± 0.006 min^−1^ and *k*_on_ = 0.180 ± 0.072 min^−1^ with solution 1, while *k*_off_ = 0.011 ± 0.004 min^−1^ and *k*_on_ = 0.113 ± 0.034 min^−1^ with solution 2. In solutions 1 and 2, it shows that the averaged *k*_on_ increased more than 10 times compared to *k*_off_. The rate ratios of each AuNR were also calculated. Fig. 3D shows the histogram of the rate ratio with solution 1 (blue), solution 2 (green), and solution 3 (magenta). The average rate ratios are 13.46 ± 6.94 and 12.08 ± 5.17 for solutions 1 and 2, respectively, demonstrating that the growth rates increase more than 12 times under interband excitation with PVP regardless of the presence of the hole scavenger. No enhanced growth rate is observed when excited by interband transition without PVP as the rate ratio for solution 3 is 0.95 ± 0.57. As the hole scavenger separates the electron–hole pair and promotes the hot-electron lifetime, we proposed that PVP rather than ethanol induces electron–hole separation. Indeed, the HOMO of PVP is −5.93 eV,^63^ which is higher than the onset of the Au d-band (∼−7.6 eV) and lower than the Fermi level of Au (−5.2 eV). The interband excitation creates electrons and holes populating near the Fermi level and d-band, respectively. The d-band holes subsequently transfer to the HOMO of PVPs, leading to the charge separation at the Au–PVP interface and enhancing the lifetime of the electron–hole pairs. This process is efficient because PVP ligands are adsorbed on the Au surface. The long-lived electrons on the Au surface reduce Au^3+^ to deposit Au atoms on AuNRs for growth. Therefore, efficient charge separation by PVP is responsible for the enhanced growth rate. The holes in PVP ligands further oxidize ethanol or water to prevent electron–hole recombination. This is evidenced by a slightly higher enhanced growth rate with ethanol, which more effectively removes holes on PVPs to inhibit the recombination of holes and electrons. Furthermore, no enhanced growth was observed in the presence of ethanol but without PVP because of the lack of hole quenching, as ethanol needs to diffuse to the Au surface to remove holes before electron–hole recombination.

**Fig. 4:**
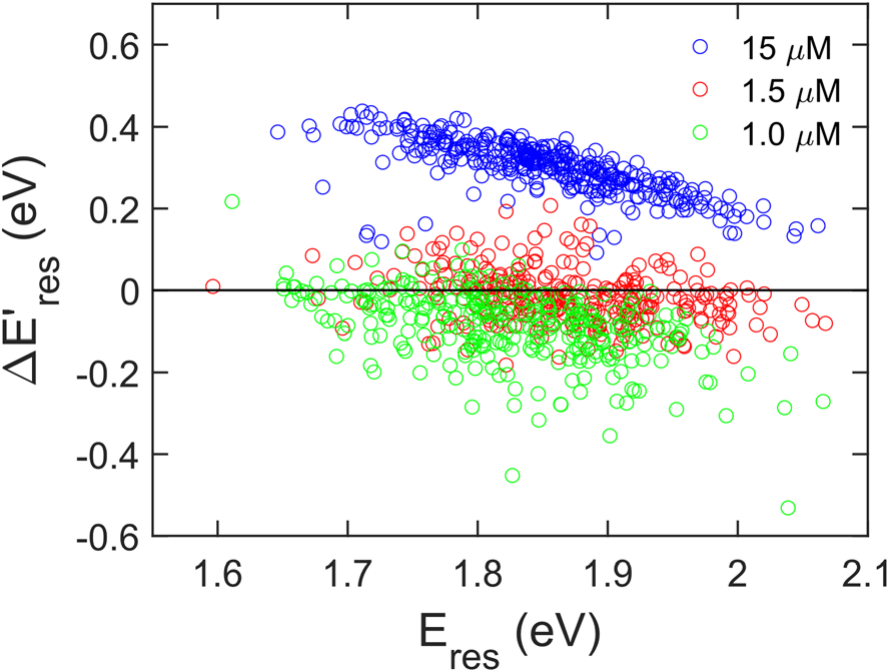
The change of *E*_res_ (
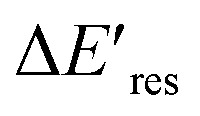
) as a function of *E*_res_ at *t* = 0 min with 15 μM (blue), 1.5 μM (red), and 1.0 μM (green) PVP in the growth solution. The black line indicates 
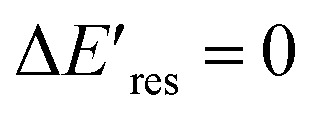
.

Next, we controlled the adsorption position of PVP on AuNRs to identify the location of electrons after the interband excitation. The Liz-Marzán group has shown that PVP ligands preferentially bind to the {111} facet, which is located at the tip of AuNRs, compared to {110} and {100}.^64^ Therefore, PVP ligands are adsorbed at the tip of AuNRs with a low PVP concentration, while uniformly covering AuNRs at a high PVP concentration. If the electrons localize near PVP after charge separation, the Au^3+^ reduction should occur at the tip of AuNRs, leading to a redshift of *E*_res_ due to a larger aspect ratio. In contrast, if the electrons delocalize in AuNRs after the holes transfer to PVP, a blueshift of *E*_res_ is expected because Au^3+^ is reduced at a random position, causing an isotropic growth. To prove this concept, we performed experiments with the growth solution containing 3 μM HAuCl_4_ and 2.9 M ethanol with various concentrations of PVP. The reduction reaction was carried out in the dark at *t* = 0–30 min, and then under the illumination of blue light at *t* = 40–60 min. Fig. 4 shows 
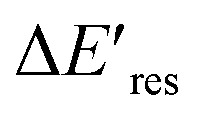
*versus E*_res_ at *t* = 0 min, where 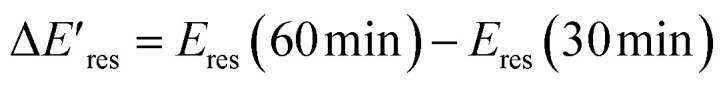 representing the difference in peak energy after illuminating blue light for 30 min. For the PVP concentration of 15 μM (blue), 
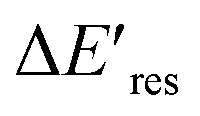
 is positive for all AuNRs, indicating a blueshift of *E*_res_ with interband excitation. Interestingly, approximately 63% of AuNRs show a negative 
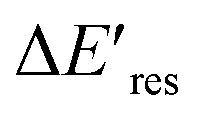
 (redshift) when changing the PVP concentration to 1.5 μM (red). Further reducing the concentration to 1.0 μM leads to more redshift in *E*_res_ (green), with 88% of AuNRs showing a negative 
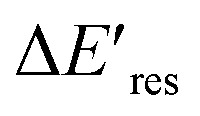
. Because PVP ligands preferentially adsorb at the tip of AuNRs at low PVP concentration, the redshift of *E*_res_ arises from the deposition of Au atoms at the tip of AuNRs, implying the localized electrons at the Au–PVP interface after electron–hole separation. With a high concentration of PVP, the AuNRs are covered with PVP ligands, and the electrons are evenly distributed on the AuNR surface after charge separation, causing isotropic growth, which leads to a blueshift of *E*_res_. A schematic illustration of this mechanism is depicted in Fig. S13.† All these results strongly indicate that the electrons are localized at the Au–PVP interface after charge separation.

**Fig. 5:**
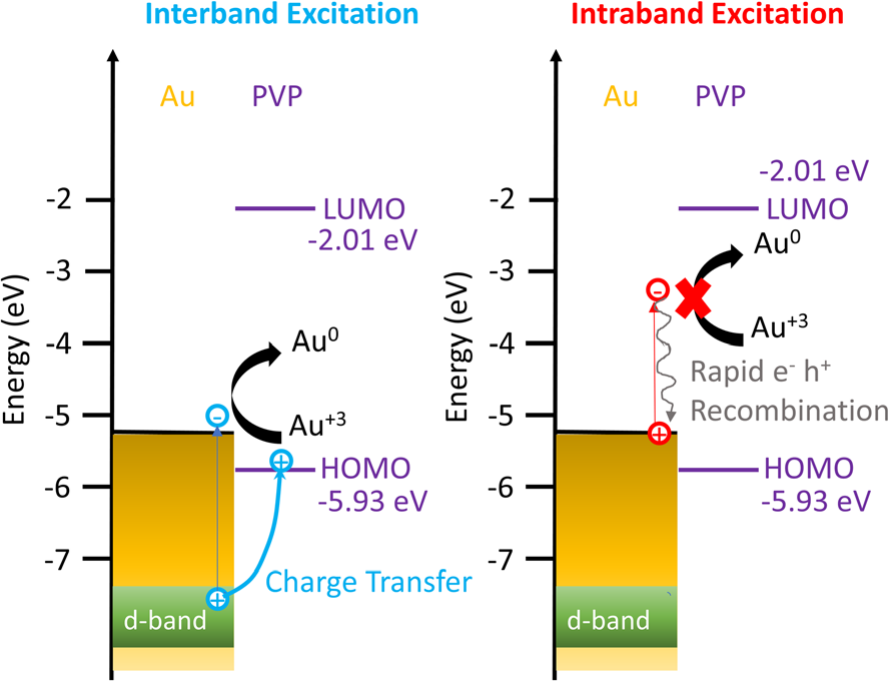
Energy diagram showing the mechanism of enhanced Au^3+^ reduction driven by interband excitation and no enhancement of Au^3+^ reduction excited by the intraband transition.

Based on these results, we propose the mechanisms of the photocatalytic reduction of Au^3+^ on AuNRs with PVP, as depicted in Fig. 5. When excited with an interband transition, it creates d-band holes and sp-band electrons, which populate near the Fermi level. The d-band holes at −7.6 eV subsequently transfer to the HOMO of PVP at −5.93 eV as the holes move upward in energy spontaneously, leading to charge separation to prolong the lifetime of the hot carriers. The electrons at the Au–PVP interface reduce Au^3+^ to induce AuNR growth, while the holes in PVP undergo an ethanol or water oxidation reaction. On the other hand, the intraband excitation generates electrons populating at the states with energy equivalent to the photon energy above the Fermi level and the holes near the Fermi level. The energy of holes is higher than that of the PVP HOMO. Additionally, the LUMO of PVP locates at −2.01 eV, which is ∼3.2 eV higher than the Fermi level of Au. As the energy of longitudinal plasmons is less than 2 eV, the intraband excitation could not promote the electron population from the Fermi level of Au to the LUMO of PVP. Therefore charge separation is forbidden under the excitation of intraband transition. Then electron–hole recombination occurs before the electrons reduce Au^3+^. As a result, no photocatalytic reduction was observed under the intraband transition. It is worth noting that the activities of plasmonic photocatalysts behave linearly with excitation power under interband and intraband transitions.^54,55^ The growth rate of AuNRs is expected to increase linearly with the excitation power excited by the interband transition. However, increasing the illumination power for the intraband transition should have a minimum impact on the growth rate due to ultrafast electron–hole recombination. Photoexcitation of plasmonic nanoparticles elevates the temperature on the nanoparticle surface and enhances the catalytic activities. Given the lower excitation power in our experiment, the temperature only increases less than 1 °C on the nanoparticle surface, and therefore the increased temperature due to the photoexcitation has no impact on the growth rate, as observed in the inset of Fig. 2D.

The results of this study can be expanded to plasmonic nanoparticles with various materials and morphologies. The HOMO of PVP is located at −5.93 eV, which is higher than the d-band of silver (Ag) and copper (Cu), located at approximately −8.2 eV and −6.6 eV, respectively.^36^ Charge separation occurs at the metal–PVP interface upon photoexciting the interband transition for Au, Ag, and Cu. Additionally, the interband transition is determined by the bulk materials and is independent of the morphology of nanoparticles. As a result, our findings can be applied to noble nanoparticles of any shape.

## Conclusion

In summary, using single-particle DFS spectroscopy, we have demonstrated PVP-induced charge separation to enhance the catalytic activity of the Au^3+^ reduction reaction under interband excitation. Probing the spectral evolution of single AuNRs in the presence of PVP ligands confirms that the AuNRs transform into large and isotropic nanoparticles excited by the interband transition. The growth rate increases 13 times under interband excitation compared to without illumination. The intraband transition does not contribute to the Au^3+^ reduction. PVP ligands rather than hole scavengers are critical to enhancing the reduction reaction. These results elucidate that PVP induces electron–hole separation with interband excitation to prolong the lifetime of sp-band electrons, which subsequently reduce Au^3+^. The hot carriers generated by the intraband excitation suffer from ultrafast electron–hole recombination, prohibiting the reduction reaction. This study provides an alternative method to utilize capping ligands to promote the photocatalysis of plasmonic nanoparticles.

## Data availability

Materials, protocols for sample preparation and single-particle measurements, SEM images of AuNRs and Au nanoparticles, the extinction spectrum of AuNR solution, color images of AuNRs during the reduction reaction, results of a control experiment using CTAB-functionalized AuNRs, and detailed data analysis for single-particle measurements can be found in the ESI.

## Author contributions

W. S. C. and B. R. conceived the idea and designed the experiments. B. R. and T. V. performed experiments and analyzed the data. W. S. C. wrote the manuscript with contributions from all authors. All authors have given the approval of the manuscript.

## Conflicts of interest

There are no conflicts to declare.

## Supplementary Material

SC-014-D3SC02167J-s001
