# Supplementary material for: Promoting plasmonic photocatalysis with ligand-induced charge separation under interband excitation
Source: Chem Sci. 2023 Jul 27;14(32):8598–606. doi: 10.1039/d3sc02167j (PMC10430595; doi:10.1039/d3sc02167j)
Supplement: SC-014-D3SC02167J-s001 [file SC-014-D3SC02167J-s001.pdf]

Electronic Supplementary Material (ESI) for Chemical Science.
This journal is © The Royal Society of Chemistry 2023

Supplementary Information

Promoting Plasmonic Photocatalysis with Ligand-Induced Charge Separation Under Interband Excitation

Ben Roche[1§], Tamie Vo[1§], and Wei-Shun Chang[1]*

[1]Department of Chemistry and Biochemistry, University of Massachusetts Dartmouth, North Dartmouth, Massachusetts, United States, 02747

[§] These two authors contribute equally.

[*]Corresponding author: Wei-Shun Chang, email: wchang2@umassd.edu

Keywords: plasmon, photocatalysis, charge separation, hot carriers, polyvinylpyrrolidone

Methods:

Material. PVP-capped AuNRs and CTAB-coated AuNRs were purchased from Nanopartz. Gold (III) chloride trihydrate ($HAuCl_4.3H_2O$, 99.9%, 520918-1G) and polyvinylpyrrolidone (PVP, M.W.= 40,000, PVP40-50G) were purchased from Sigma-Aldrich. Potassium hydroxide (KOH, P250-1), reagent alcohol (89 to 91%, A995-4), acetone (99.5%, A929-4), hydrochloric acid (6N), hellmanex® III, and absolute ethanol (molecular biology grade, 99.5%, BP2818-500) were purchased from Fisher Scientific. 3-aminopropyltriethoxysilane (APTES, >98%, A0439) was purchased from TLI America. Deionized (DI) water was used in all experiments.

Preparation of silanized coverslip. Glass coverslips (24 mm x 60 mm No. 1.5, Thermo Scientific) were cleaned by sonicating in hellmanex (2%), reagent alcohol, acetone, KOH (1M), and DI water. The cleaned coverslips were sonicated in acetone for 5 minutes and dried with N_2 flow. Then, the coverslips were submerged in a 1% APTES solution for 3 minutes, immediately rinsed with 10 ml of acetone, and dried with N_2.

Preparation of flow cells. Two holes were drilled in glass slides (25 x 75 x 1 mm, Fisherfinest), which were cleaned by sonicating in hellmanex (2%), reagent alcohol, acetone, KOH (1M), and DI water. The diluted PVP-capped AuNR solution was spin-coated onto the silanized coverslip, which was assembled into a flow cell with a glass slide spaced by a parafilm. To seal the cell, the flow cell was placed on a hot plate at 140 °C for a few seconds to melt the parafilm. The two tubes (Scientific Commodities, BB31695-PE/4) were connected to the holes through tubing connectors (Grace Bio-Labs, 460003) and sealed with epoxy glue.

Single-particle DFS spectroscopy. Single-particle DFS measurements were taken with a custom-built DFS microscope consisting of an inverted microscope (Zeiss, Axio-observer 5) and a spectrometer (Princeton Instrument, ARC-SP-2156) equipped with a CCD camera (Princeton Instrument, PIX-400BRX) mounted on a motorized linear stage (Newport, CONEX-LTA-HL). Essentially, white light from a halogen lamp (100W) was focused on the sample through a dark-field condenser (N.A. = 1.4). The scattered light of AuNRs was collected using an oil immersion objective (Zeiss, PlanAchrmat, N.A. = 0.7, 63 ×), directed to the spectrometer located at the first image plane of the microscope. Hyperspectral imaging was achieved by moving the slit of the spectrometer across the scattering image while taking the spectra. A custom-written Labview program was used to control the linear stage and the spectrometer. The analyses of the hyperspectral images were carried out by a custom-written Matlab program. The reported spectra were background subtracted and normalized by the lamp spectra. Although the intensities of scattering spectra are reported in an arbitrary unit, the spectral intensities of the nanoparticles in the same cell can be directly compared, considering that our hyperspectral images were acquired under identical conditions.

Procedure for acquiring the spectral evolution of AuNRs during the reaction. The sequence of the experiment is shown in Figure S4. DI water was first introduced to the flow cell (first valley of the blue line), and the hyperspectral image of AuNRs was acquired (first color bar). We define the reaction time for this image as 0 minute, t = 0 min, as no reaction occurred. Subsequently, the reagent containing 3 μM $HAuCl_4$, 15 μM PVP, and 2.9 M ethanol was flown

into the cell and incubated for 10 minutes to allow the reduction reaction of Au^{+3} to deposit on AuNRs without light illumination (first peak of the blue line). The reaction was stopped by flowing DI water into the cell to remove the reactants completely (second valley of the blue line). Then another hyperspectral image of the identical region was acquired (second color bar). The reaction time was 10 minutes (t = 10 min) because AuNRs were incubated in the reagent for 10 minutes. The sequences of the introduction of reagent and DI water (peak and valley of the blue line in Figure S4) were repeated, followed by the acquisition of hyperspectral images (color bars in Figure S4) to obtain AuNR spectra at t = 20, 30, 40, and 50 min without light illumination. For the photoinduced reduction reaction, the reagent was flown into the cell with an incubation time of 10 minutes while the lamp was turned on (the first peak of the golden line). After removing the reagent, hyperspectral imaging of the same location was measured at t = 60 min. The same procedures were repeated five times to obtain AuNR spectra at t = 70, 80, 90, 100, and 110 min. The excitations of interband and intraband transitions were realized by placing a blue filter (500 nm short-pass filter) and a red filter (610 nm long-pass filter) after the lamp, respectively. Two IR filters were also inserted in the excitation path to avoid heating the aqueous solution by absorbing IR photons. The power of excitation light was measured using a thermal power meter (Thorlabs, S405C). The power densities for interband and intraband excitations were 5 and 40 W/cm^2, respectively.

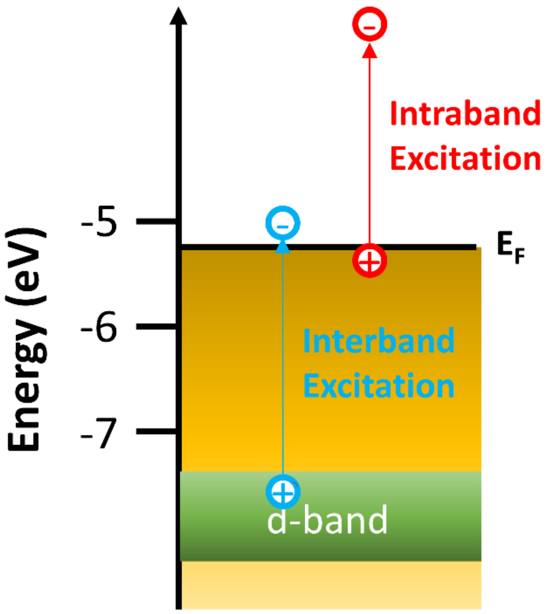

Figure S1. Schematic illustration of the excitation of interband and intraband transitions of AuNRs.

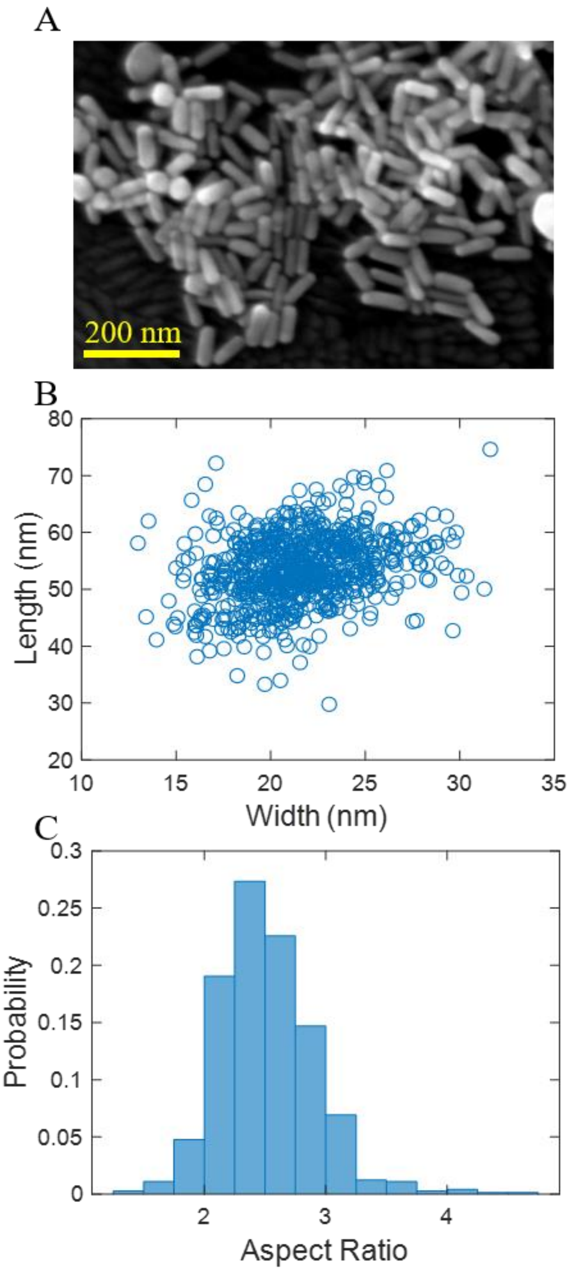

Figure S2. Morphologies of AuNRs. (A) Representative SEM image of AuNRs. Scale bar: 200 nm. (B) Scatter plot of the widths and lengths of AuNRs. The width and lengths of the AuNRs are 21.6 ± 3.1 and 53.5 ± 5.9 nm, respectively, averaged from 735 AuNRs. (C) Histogram of the aspect ratio of AuNRs. The aspect ratio is 2.5 ± 0.4, averaged from 735 AuNRs.

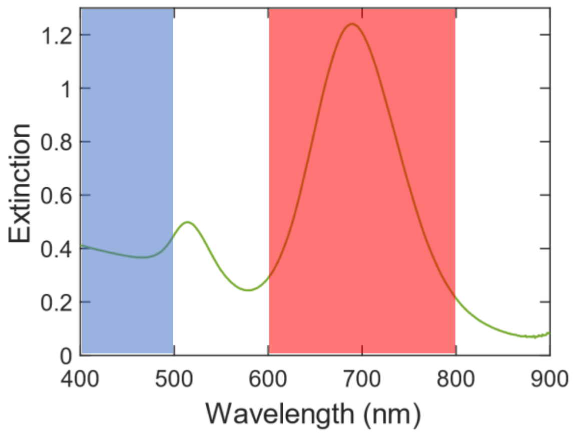

Figure S3. Extinction spectrum of the AuNR solution. The blue and red areas represent the spectral range of blue and red filters, respectively.

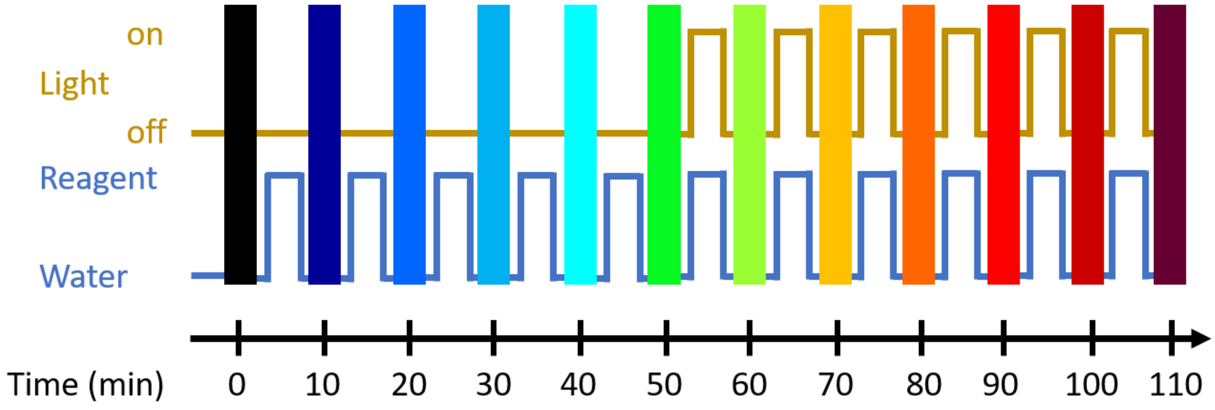

Figure S4. Sequence of the experiment. The blue line represents the injection of water (valley) and reagent (peak) into the cell. The golden line indicates the light on (peak) and off (valley). The color bars are the sequence of hyperspectral images. The time duration of the peak for the blue and golden lines is 10 minutes.

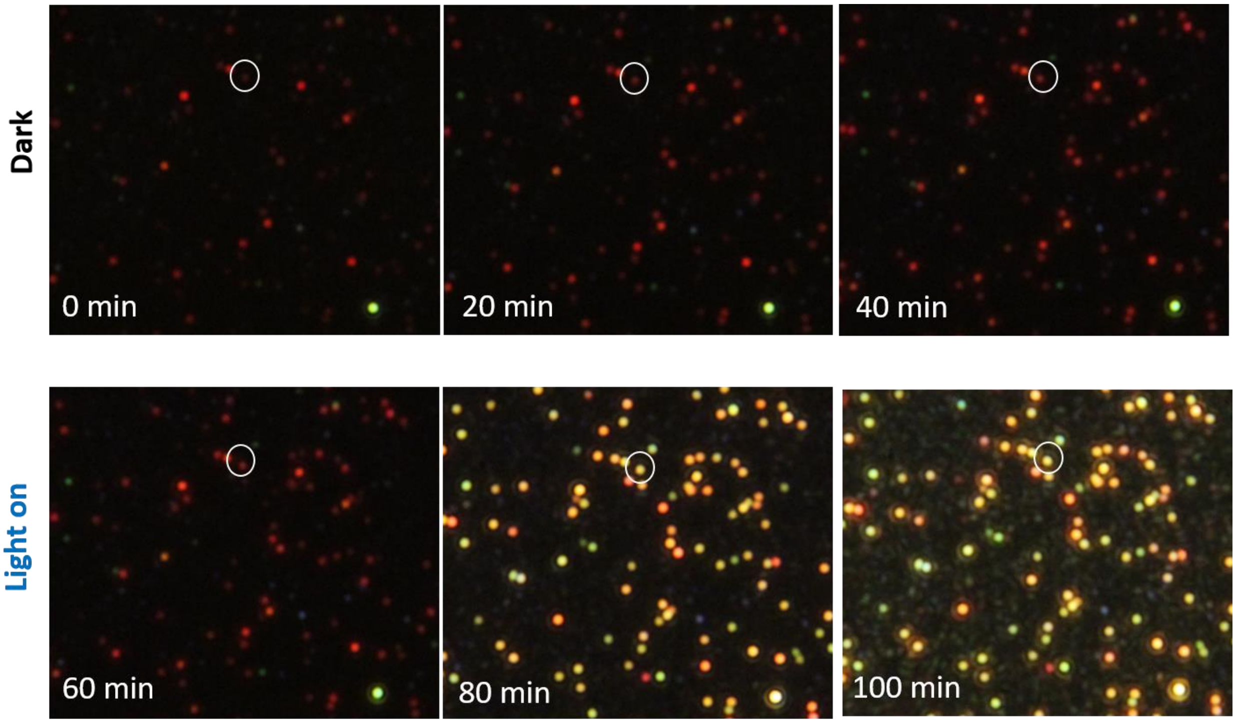

Figure S5. Color images of the AuNRs as a function of reaction time. The top and bottom rows of the images were taken for the reactions in the dark and illuminated by blue light, respectively. The white circle indicates the AuNR with the spectral evolution shown in Figure 1D. The color of AuNR changes from dim red to bright yellow, suggesting the reshaping of AuNR into a large isotropic nanoparticle. The photos were taken with a single-lens reflex camera after acquiring the hyperspectral images.

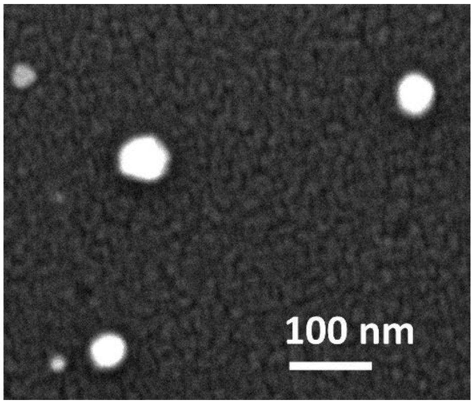

Figure S6. Representative SEM image of isotropic nanoparticles transformed from AuNRs after being illuminated with blue light for 110 minutes in the reaction. Scale bar: 100 nm.

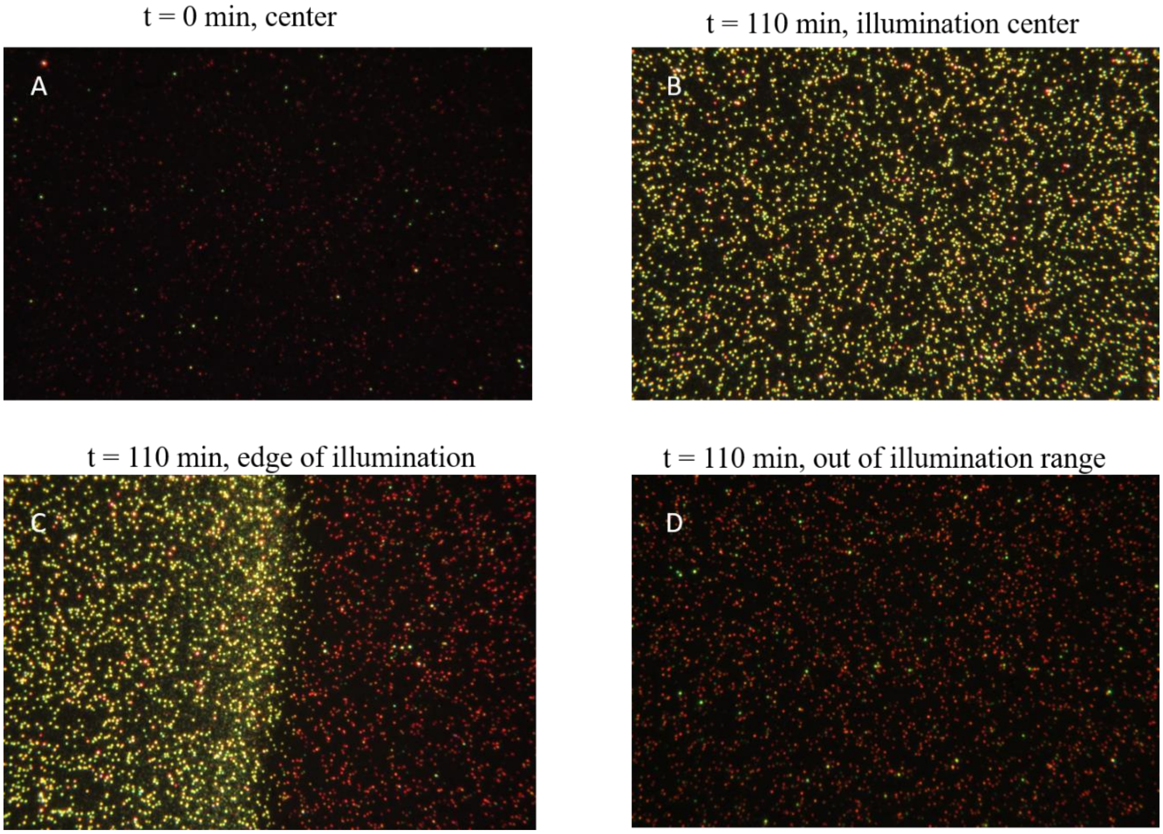

Figure S7. Color images of AuNRs at t = 0 min (A) and t = 110 min (B) at the same location. (C) Color image of AuNRs at the illumination boundary at t = 110 min. The AuNRs turn into yellow and green colors with higher intensities under blue light illumination, while AuNRs remain red color without the illumination. (D) Color image of AuNRs outside the illumination area. The red color of the particles suggests no transformation of AuNRs into an isotropic shape.

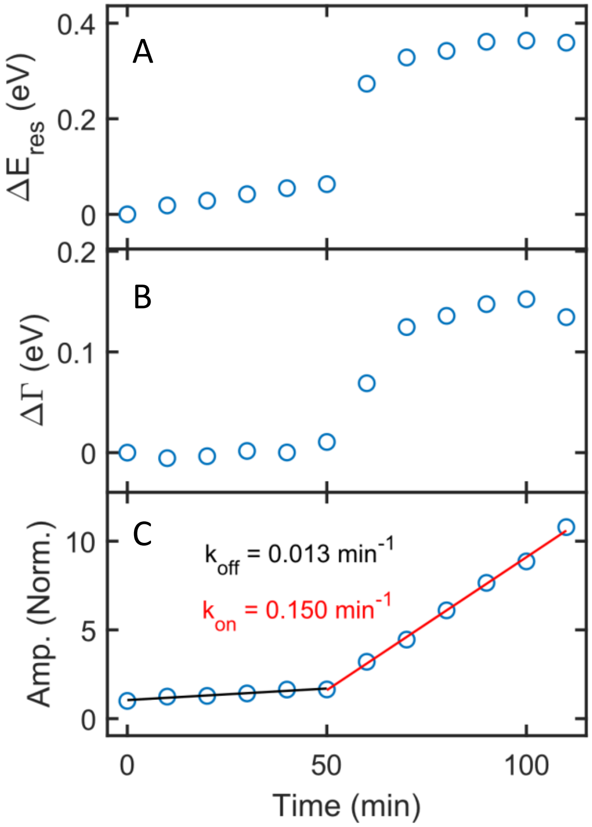

Figure S8. Changes in E_{res} (ΔE_{res}, A) and Γ (ΔΓ, B), and normalized amplitude (C) as a function of the reaction time. ΔE_{res}, ΔΓ, and normalized amplitude is calculated as $\Delta E_{res}(t) = E_{res}(t) - E_{res}(0)$, $\Delta\Gamma(t) = \Gamma(t) - \Gamma(0)$, and $\text{Normalized Amplitude } (t) = \text{Amplitude}(t)/\text{Amplitude}(0)$, respectively. The time-dependent normalized amplitude in the dark and under illumination is also fit to a linear equation ($y = kt + b$, where t is the reaction time, k is the rate constant, and b is a constant) to get the apparent rate constant in the dark (k_{off}) and under illumination (k_{on}), respectively.

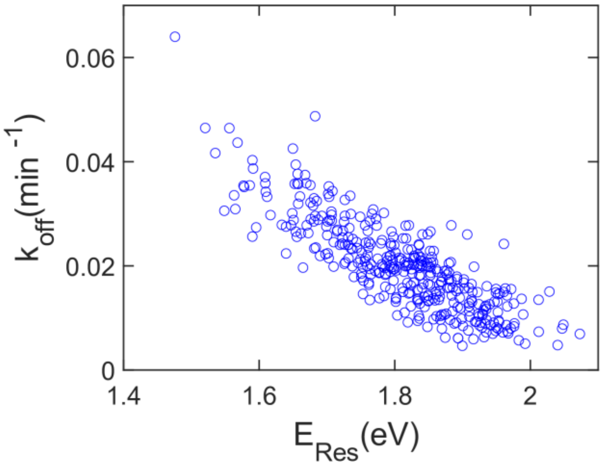

Figure S9. Correlation between k_{off} and E_{res} at t = 0 min. E_{res} at t = 0 min represents the initial aspect ratio of AuNRs before the reaction. The AuNRs with a larger aspect ratio show low energy for E_{res}. The correlation indicates that AuNRs with a larger aspect ratio possess a higher growth rate in the dark.

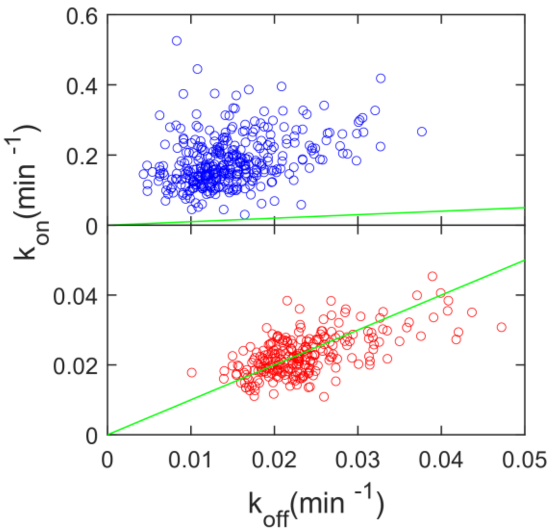

Figure S10. Correlation between k_{off} and k_{on} for interband (top) and intraband (bottom) transitions. The green lines represent the one-to-one correlation.

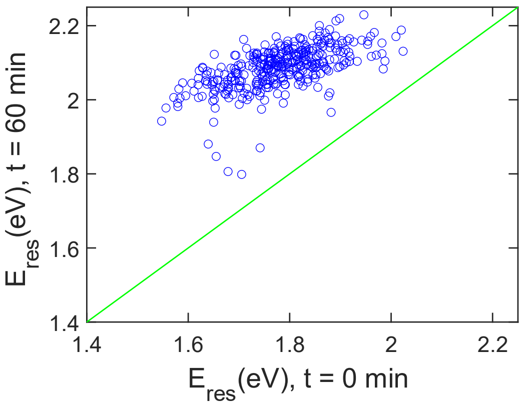

Figure S11. Correlation between E_{res} of AuNRs at t = 0 and 60 min. E_{res} at t = 60 min for most AuNRs shift to > 2.0 eV, suggesting that AuNRs are transformed into an isotropic shape after interband excitation for 10 minutes. The green lines represent the one-to-one correlation.

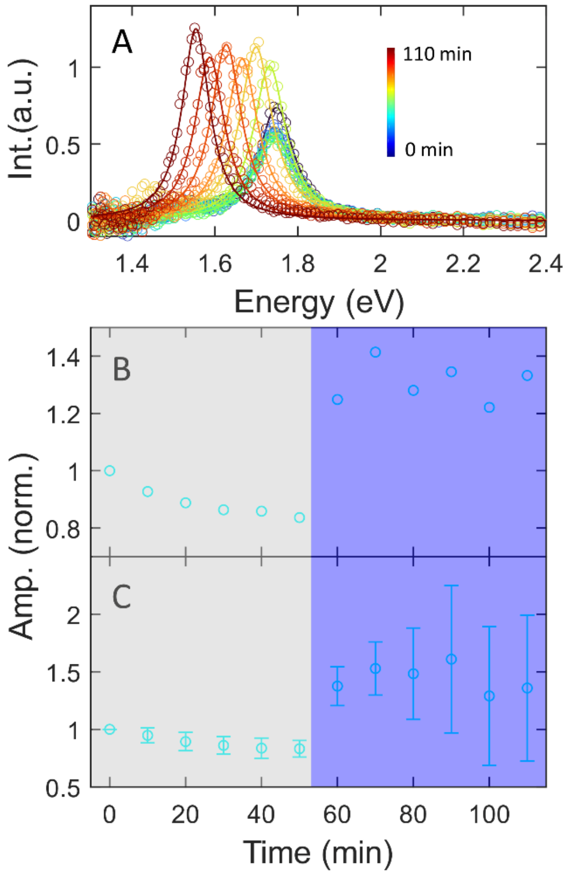

Figure S12. Spectral change of CTAB-functionalized AuNRs in growth solution 3 with interband excitation. (A) Spectral evolution of a representative CTAB-functionalized AuNR in growth solution 3 with interband excitation. (B) Temporal evolution of the normalized amplitude of the scattering spectra shown in (A). The grey and blue shadows indicate the blue light off and on, respectively. (C) Subensemble average of the normalized amplitude of AuNRs in growth solution 3 under interband excitation. The symbols and error bars represent the mean and standard deviation of the normalized amplitudes of AuNRs at a specific reaction time.

In the dark, the normalized amplitude decreases with reaction time, probably due to the dissolution of silver layers on CTAB-functionalized AuNRs, consistent with the previous study.[1] When illuminated with blue light, the normalized amplitude increases slightly and fluctuates with reaction time. Therefore, the amplitude evolution cannot fit with the linear equation to obtain the rate ratio with light on and off. The small increase in the normalized amplitude indicates no significant growth of AuNRs in the absence of PVP with interband excitation.

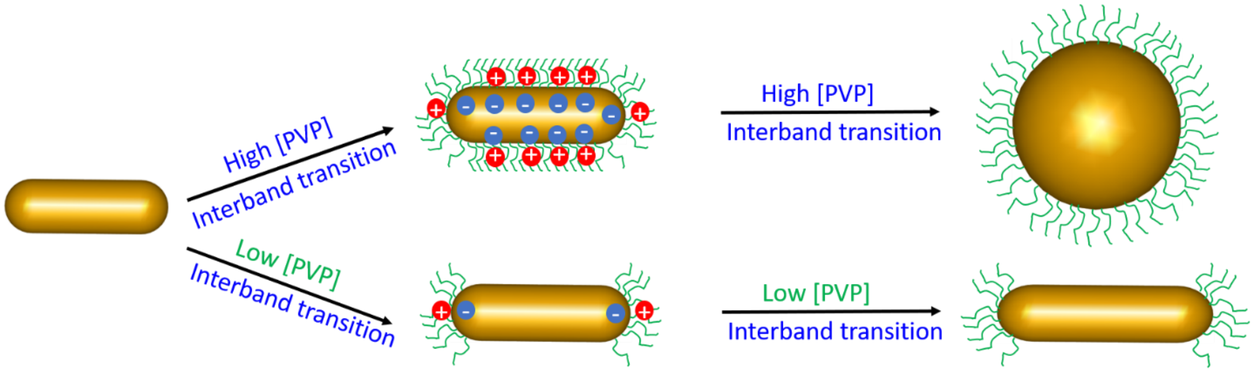

Figure S13. A schematic illustration of AuNR growth at different PVP concentrations.

References:

1. W. Ye, K. Krüger, A. Sánchez-Iglesias, I. García, X. Jia, J. Sutter, S. Celiksoy, B. Foerster, L. M. Liz-Marzán, R. Ahijado-Guzmán and C. Sönnichsen, *Chem. Mater.*, 2020, **32**, 1650-1656.
